# Carrier Multiplication in Transition Metal Dichalcogenides Beyond Threshold Limit

**DOI:** 10.1002/advs.202203400

**Published:** 2022-09-07

**Authors:** Yuxiang Liu, Thomas Frauenheim, ChiYung Yam

**Affiliations:** ^1^ Bremen Center for Computational Materials Science University of Bremen Am Fallturm 1 28359 Bremen Germany; ^2^ Beijing Computational Science Research Center Haidian District Beijing 100193 China; ^3^ Shenzhen JL Computational Science and Applied Research Institute Shenzhen 518109 China; ^4^ Shenzhen Institute for Advanced Study University of Electronic Science and Technology of China Shenzhen 518000 China; ^5^ Hong Kong Quantum AI Lab Limited Hong Kong 0000 China

**Keywords:** 2D materials, carrier multiplication, chalcogen vacancy, non‐adiabatic molecular dynamics, time‐dependent density‐functional theory

## Abstract

Carrier multiplication (CM), multiexciton generation by absorbing a single photon, enables disruptive improvements in photovoltaic conversion efficiency. However, energy conservation constrains the threshold energy to at least twice bandgap (2$E_{g}$). Here, a below threshold limit CM in monolayer transition metal dichalcogenides (TMDCs) is reported. Surprisingly, CM is observed with excitation energy of only 1.75 $E_{g}$ due to lattice vibrations. Electron–phonon coupling (EPC) results in significant changes in electronic structures, which favors CM. Indeed, the strongest EPC in monolayer MoS_2_ leads to the most efficient CM among the studied TMDCs. For practical applications, chalcogen vacancies can further lower the threshold by introducing defect states within bandgap. In particular, for monolayer WS_2_, CM occurs with excitation energy as low as 1.51 $E_{g}$. The results identify TMDCs as attractive candidate materials for efficient optoelectronic devices with the advantages of high photoconductivity and efficient CM.

## Graphical abstract

Lattice vibrations induce reduction of bandgap in transition metal dichalcogenides. The effect overshadows phonon‐assisted relaxation of carriers and results in carrier multiplication below the threshold energy limit.

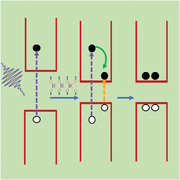

## 1 Introduction

Photo‐excitation of semiconductors with photon energies exceeding their bandgap ($E_{g}$) creates electron‐hole pairs that can be collected to produce an electric current. When the excess energy of photoexcited carriers is higher than $E_{g}$, carriers obtain sufficient energy to excite another electron‐hole pair, a process that is known as carrier multiplication (CM). Beard et al. predicted that CM could overcome the Schokley–Queisser limit and raise solar cell efficiency to ∼ 46%.^[^ ^1^, ^2^, ^3^ ^]^ For maximal use of energy, with energy conservation limit and competition from phonon‐assisted relaxations, the threshold energy of CM should be closed to ∼ 2 – 2.5 $E_{g}$.^[^ ^4^ ^]^ However, in bulk semiconductors, the threshold energy can reach as high as ∼ 6 $E_{g}$,^[^ ^5^ ^]^ and renders its application impractical. This is attributed to the relatively weak Coulomb interactions and the constraints imposed by momentum conservation. In addition, phonon scatterings act as a competing pathway that efficiently dissipates the excess energy. As a result, CM is rarely realized in photovoltaic devices based on traditional bulk materials. In contrast, CM performance can be enhanced in nanostructures, where quantum confinement relaxes the strict momentum conservation requirement and phonon bottleneck inhibits phonon emission.^[^ ^6^, ^7^, ^8^, ^9^ ^]^ It has been reported that CM threshold energy in bulk PbSe and PbS is as high as 4.5 $E_{g}$ whereas it is substantially reduced to 2.7 and 3 $E_{g}$ in corresponding quantum dots^[^ ^4^ ^]^ and 2.2 $E_{g}$ in nanorods.^[^ ^10^ ^]^ Recently, CM phenomenon is observed in 2D transition metal dichalcogenides (TMDCs) films of 2H‐MoTe _2_ and 2H‐WSe _2_ with threshold energy as low as 2 $E_{g}$, and conversion efficiency can reach nearly 99%.^[^ ^11^, ^12^ ^]^ These characteristics are superior to those previously reported nanostructured materials.^[^ ^13^, ^14^, ^15^, ^16^, ^17^, ^18^ ^]^ To further optimize materials for photovoltaic applications, it is interesting to explore if the threshold energy limit can be overcome.

2D TMDC materials, represented by MX_2_ (M = Mo, W; X = S, Se, Te), are intensively investigated owing to their fascinating properties. In particular, MX_2_ exhibits indirect‐to‐direct bandgap transition when exfoliated from bulk to monolayer.^[^ ^19^, ^20^ ^]^ The weak van der Waals interaction between individual layers allows easy formation of multiple layered structures of different materials.^[^ ^21^ ^]^ These heterostructures permit further tuning of material properties. MX_2_ also presents distinctive electrical and optical properties. It is demonstrated that the carrier mobility of monolayer MX_2_ is at least 200 cm^2^V^−1^s^−1^ at room temperature, similar to graphene nanoribbons.^[^ ^22^ ^]^ The mobility of WSe_2_ can reach up to 500 cm^2^V^−1^s^−1^ that is comparable to the best performance of single‐Si crystal.^[^ ^23^, ^24^ ^]^ In addition, the absorption of sunlight in monolayer MX_2_ is typically 5–10%,^[^ ^25^, ^26^ ^]^ an order of magnitude larger than that in common photovoltaic materials.^[^ ^27^ ^]^ Thus, exceptional mechanical flexibility, bandgap tunability, high charge‐carrier mobility, and efficient sunlight absorption, together with the possibility of exhibiting CM, render TMDCs as promising materials for highly efficient next‐generation solar cells.

In this paper, we combine nonadiabatic molecular dynamics (NAMD) with real‐time time‐dependent density functional theory (TDDFT) to investigate CM phenomenon in various monolayer TMDCs. Our results show that CM phenomenon is observed in all six monolayer TMDCs, with threshold energy as low as 1.75 $E_{g}$ due to couplings to phonons. It is found that lattice vibrations induce significant changes in electronic structures, which favors CM and overshadows the effect of phonon‐assisted carrier relaxation. This leads to occurrence of CM with excitation energy lower than 2 $E_{g}$. When the excess energy are the same in the six monolayer TMDCs, MoS_2_ with the strongest EPC achieves the highest CM conversion efficiency $\eta_{\text{CM}}$. In addition, we show that chalcogen vacancies, as a common defect in TMDCs, can also promote CM by scattering electrons from valence bands to defect states. In particular, sulfur vacancies lower the onset energy of CM to 1.51$E_{g}$ in monolayer WS_2_. This work demonstrates the role of phonon modes on CM process, and provides insight into the design strategy to break CM threshold limit in TMDCs via phonon and defect engineering.

## 2 Results and Discussions

### 2.1 Carrier Excitation Dynamics

**Figure 1:**
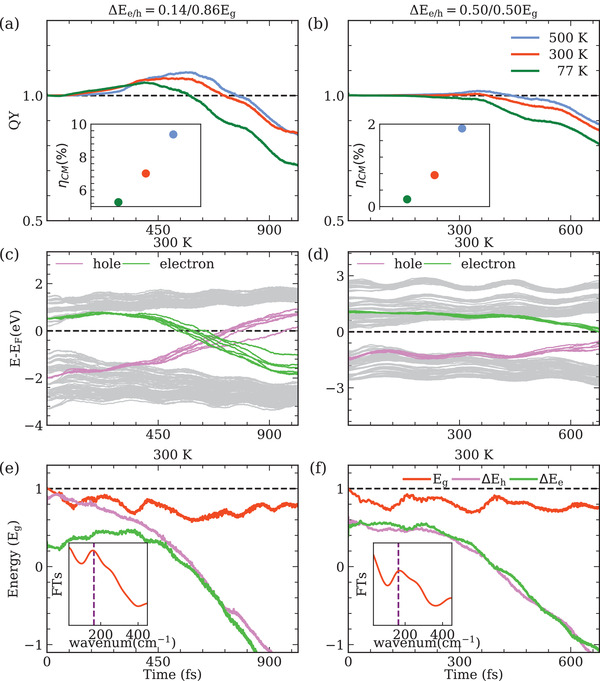
Excitation dynamics of monolayer MoTe_2_ with different carrier excess energies at different temperatures. Electron‐hole pairs are generated by photon with energy of 2$E_{g}$ at temperature 77, 300, and 500 K. The excess energies of carrier are $\Delta E_{\text{e/h}}=0.14/0.86E_{g}$ (left panel) and $\Delta E_{\text{e/h}}=0.5E_{g}/0.5E_{g}$ (right panel). a,b) Carrier generation QY of CM in MoTe_2_ as a function of time upon excitation. Inset: CM conversion efficiency at the three temperatures. c,d) Time‐evolution of energy states at 300 K. Purple and green lines are hole and electron states, respectively. The dashed line shows the Fermi level $E_{F}$. e,f) $E_{g}\left(t\right)$ at 300 K is compared with the excess energy of hole $\Delta E_{h}$ and electron $\Delta E_{e}$. The dashed line represents the bandgap at t=0 fs. Inset: FTs of time‐dependent bandgap $E_{g}\left(t\right)$. Vertical dashed line represents phonon mode A' with vibrational frequency of 163.7 cm^−1^

We first investigate CM phenomenon in monolayer MoTe _2_. Twelve electron‐hole pairs are generated by moving electrons from valence bands to conduction bands at 300 K (more details are shown in Figure S1c, Supporting Information). The excitation energy is set as $∼2E_{g}$, where the hole and electron excess energies ($\Delta E_{h}$ and $\Delta E_{e}$) are equal to 0.86 and 0.14 $E_{g}$, respectively. The excess energy is defined as the difference in energy of the carrier with respect to the band edge. To quantify the efficiency of CM, we define the carrier generation quantum yield (QY) as the number of electron‐hole pairs produced per absorbed photon. Several recent studies reported that CM is highly efficient in layered MoTe _2_, in which the onset energy of CM can be as low as $2E_{g}$ with carrier generation QY=2.^[^ ^11^, ^12^ ^]^ Ellingson et al. attributed the low onset to the asymmetric transition where the total kinetic energy is resident in only one kind of carrier and is therefore sufficient to create additional electron‐hole pair.^[^ ^9^ ^]^ In our case, $\Delta E_{h}$ (0.86 $E_{g}$) and $\Delta E_{e}$ (0.14 $E_{g}$) are insufficient to excite another electron‐hole pair across the bandgap. Surprisingly, **Figure** **1**a shows that the amount of carriers keeps increasing upon excitation and the QY reaches 1.07 at ∼550 fs. This low conversion efficiency is caused by the relatively high carrier concentration that favors Auger recombination and phonon‐assisted nonradiative recombination. As shown in Figure 1c, both excited electrons and holes decay back to valence band and conduction band at ∼ 600 fs, respectively. The dynamics of the system with only one excited electron‐hole pair is presented in Figure S4 (Supporting Information). With a lower carrier concentration, the lifetime of electron‐hole pair becomes longer and QY reach up to 2 that is consistent with experimental measurements (More details about carrier decay process are discussed in Supporting Information). To reveal the mechanism of CM in monolayer MoTe _2_, time‐evolution of bandgap are plotted in Figure 1e. Upon excitation, the bandgap of MoTe _2_ changes periodically due to the lattice vibrations. Overall, the bandgap is reduced along with the whole trajectory and even drops to 60% of its initial value. This enables excited holes to have sufficient energy to excite an electron‐hole pair across the bandgap. Figure 1e indicates that $\Delta E_{h}\left(t\right)$ surpasses the bandgap at t∼50 fs, triggering CM in the materials. Previous studies demonstrated that tensile strain results in significant changes in electronic structure of monolayer TMDCs. This can even induce semiconductor‐to‐metal transition with deformation of ∼10%.^[^ ^28^, ^29^ ^]^ In other words, besides assisting carrier relaxations, lattice vibrations can also cause significant changes in electronic structure and carrier dynamics. To further investigate the effect of phonon on the bandgap, Fourier transform (FT) of $E_{g}\left(t\right)$ is plotted in the inset of Figure 1e. The FT displays a characteristic frequency at ∼ 163.7 cm ^−1^ that corresponds to an out‐of‐plane vibrational mode (A') of tellurium atoms. Therefore, it is expected that the A' mode of MoTe _2_ has major effect on the bandgap in the time evolution. The resulting bandgap reduction is favorable for CM process, especially when the excess energy in carriers $\Delta E_{\text{e/h}}$ is below the threshold limit.

### 2.2 Phonon‐Assisted CM Beyond Threshold Limit

**Figure 2:**
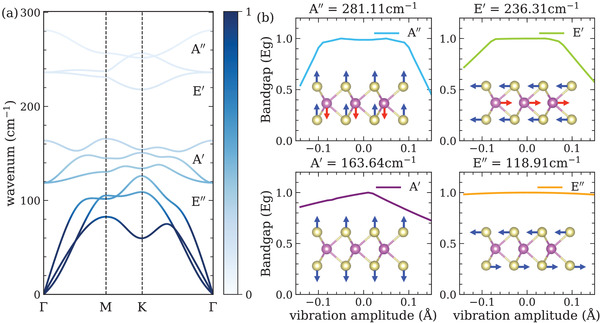
Lattice vibrational modes in monolayer MoTe_2_. a)Phonon dispersion of monolayer MoTe_2_. The colormap indicates the phonon occupation number that is determined by the Bose–Einstein distribution at 300 K. b) The bandgap as a function of the vibration amplitude of four optical phonon modes in monolayer MoTe_2_. The inset shows vibrational modes of optical phonons at Γ point.

To better understand the role of nuclear motion in CM process, the lattice vibrational modes of monolayer TMDCs are calculated using density‐functional theory (DFT) with Perdew–Burke–Ernzerhof (PBE) functional. The phonon dispersion of monolayer MoTe_2_, and ionic vibrations of four optical phonon modes are shown in **Figure** **2**. These vibrational frequencies are consistent with previous studies.^[^ ^30^, ^31^ ^]^ The effect of phonon modes on the bandgap is demonstrated in Figure 2b, where we calculate the bandgap as a function of the displacement amplitude of these modes. As shown in Figure 2b, all four optical phonons lead to narrowing of bandgap in MoTe_2_, a phenomenon that is also observed in other five TMDCs (Figures S7– S11, Supporting Information). The phonon‐dependent bandgap in our calculations are consistent with that calculated by hybrid HSE06 functional, in which the equibiaxial tensile strains can lead to phase transformation from semiconducting 2H phase to metallic 1T phase.^[^ ^28^ ^]^ As shown in the inset of Figure S12 (Supporting Information), our results also indicate that tensile and compressive strains both narrow the bandgap of monolayer MoTe_2_. Meanwhile, at room temperature, only A' and E“ modes are activated effectively according to Bose–Einstein distribution. Compared with A' mode, ionic vibrations of E mode lower the bandgap by 2% only. Thus, the contribution of E” mode can be ignored and A' mode is responsible for the reduction of bandgap at room temperature that is consistent with the results in the inset of Figure 1e.

We further study the scenario where MoTe _2_ is excited at different temperatures to demonstrate the positive effect of EPC on CM process (Figure 1). Electron‐hole pairs are generated by photons with energy of 2 $E_{g}$ at 77, 300, and 500 K, respectively. Here, we consider two types of excitation i) asymmetric electron‐hole pairs with $\Delta E_{e}=0.14E_{g}$ and $\Delta E_{h}=0.86E_{g}$ and ii ) symmetric electron‐hole pairs with $\Delta E_{e}=\Delta E_{h}=0.5E_{g}$. To facilitate our analysis, we quantify the CM conversion efficiency as follows:

(1) $$\eta_{CM}=\frac{\phi_{\max}-1}{\frac{h\nu }{E_{g}}-1}\times 100\%$$

where ϕ_max_ is the maximum QY in the time evolution, and hν is the energy of photons. In previous works, it was reported that carrier relaxation via phonons is the main channel for energy dissipation.^[^ ^4^ ^]^ Therefore, it is generally anticipated that weakening EPC is a straightforward way to promote $\eta_{\text{CM}}$. However, the situation might change if the influence of nuclear motion on electronic structure is taken into account. As shown in Figure 1a, with phonon‐induced narrowing of bandgap, CM phenomenon is observed even when the excess energy of holes $\Delta E_{h}$ is only 0.86 $E_{g}$. At a higher temperature, the vibrational excursions of atoms are larger. This results in a narrower bandgap, and consequently a higher carrier generation QY in MoTe _2_ is achieved. The CM conversion efficiency $\eta_{\text{CM}}$ is enhanced from 5.26% to 9.37% when the temperature is raised from 77 to 500 K. Similarly, increasing temperature has the same effect on improving $\eta_{\text{CM}}$ for the symmetric electron‐hole pair excitation (Figure 1b). In particular, at 77 and 300 K, CM is not observed, and nonradiative and Auger recombination dominate the carrier relaxation process. This can be explained by the fact that both $\Delta E_{e}$ and $\Delta E_{h}$ are below $E_{g}\left(t\right)$ along the whole trajectory, as shown in Figure 1f and Figure S6 (Supporting Information). However, at 77 K, only a few phonons are excited and $E_{g}\left(t\right)$ is much larger than the energy of excited carriers. On the contrary, at 500 K, intense ionic vibrations shrink the bandgap by ∼50% and trigger the onset of CM. From the FTs of E ${}_{g}$ (t) at different temperatures, as plotted in the insets of Figure 1e,f and Figures S5 and S6 (Supporting Information), it is shown that the A' mode is the main contribution to the oscillation of $E_{g}\left(t\right)$ in monolayer MoTe _2_. To further demonstrate the role of bandgap in CM process, we stimulate the carrier dynamics in monolayer MoTe _2_ under ± 3% strain. As shown in the inset of Figure S12 (Supporting Information), the bandgap of monolayer MoTe _2_ decreases significantly under both tensile and compressive strain that is expected to improve CM performance. Its effect on CM is simulated and plotted in Figure S12 (Supporting Information). Obviously, compared with the case without strain, QY of CM is enhanced, especially for the case with ‐3% strain, QY is enhanced by nearly 30% with a value of ∼ 1.12. In this way, we show the possibility to reduce the threshold energy for CM in 2D TMDCs.

**Figure 3:**
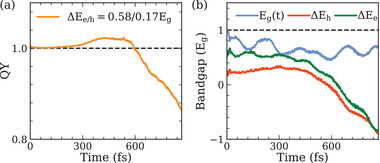
Excitation dynamics of monolayer MoTe_2_ with excitation energy of 1.75$E_{g}$. The hole and electron excess energies are $\Delta E_{\text{e/h}}$=0.58/0.17$E_{g}$. a) Carrier generation QY as a function of time. b) Time‐evolution of bandgap $E_{g}\left(t\right)$, hole excess energy $\Delta E_{h}\left(t\right)$ and electron excess energy $\Delta E_{e}\left(t\right)$.

To examine the above mechanism, we explicitly excite a monolayer MoTe _2_ with a below threshold energy of 1.75 $E_{g}$ at 300 K. Specifically, $\Delta E_{e}$ and $\Delta E_{h}$ are 0.58 and 0.17 $E_{g}$, respectively. As shown in **Figure** **3**, CM phenomenon is indeed observed after excitation and CM conversion efficiency $\eta_{\text{CM}}$ reaches ∼ 4% at 500 fs. Figure 3b shows that phonon‐induced reduction of bandgap is $∼0.45E_{g}$, thus excited electrons have sufficient energy to scatter another pair of carriers across the bandgap at ∼ 150 fs, leading to the onset of CM. The result shows the first time CM process occurs with excitation energy less than  2*E*_g_, which indicates that nuclear vibrations have a positive effect on CM process for monolayer TMDCs. Next, we further explore the possibility of phonon‐assisted CM in other MX _2_ and compare their conversion efficiencies $\eta_{\text{CM}}$.

**Figure 4:**
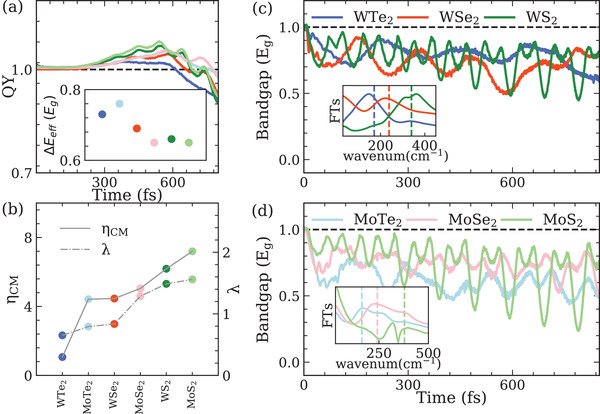
Excitation dynamics of the six monolayer TMDCs. Electron‐hole pairs are excited with photon energy of 2 $E_{g}$, with excess energies distributed as $\Delta E_{\text{e/h}}∼0.7/0.3E_{g}\left(\mathrm{or}∼0.3/0.7E_{g}\right)$. a) Time‐evolution of carrier generation QY of CM. The inset presents the effective carrier excess energy Δ E ${}_{eff}$ in six monolayer TMDCs. b) Comparison of CM conversion efficiency $\eta_{CM}$ and EPC strengths λ in the six monolayer TMDCs. Time‐evolution bandgap $E_{g}\left(t\right)$ of c) WX _2_ and d) MoX _2_ (X = S, Se, Te). Insets: FTs of $E_{g}\left(t\right)$. Colored dashed lines represent the frequencies of phonon mode A' in each TMDC.

The EPC strength for electronic state nk is defined as

(2) $$\lambda_{nk}=2\int d\omega \frac{\begin{array}{c} \sum_{\nu q}\sum_{n^{\prime }}{\left|g^{\nu }\left(nk,n^{\prime }k+q\right)\right|}^{2}\\ \;\times \delta \left(\varepsilon_{n^{\prime }k+q}-\varepsilon_{nk}-\hbar \omega_{\nu q}\right)\delta \left(\hbar \omega -\hbar \omega_{\nu q}\right) \end{array}}{\omega }$$

where the summations include all electron scattering events from states $\varepsilon_{n^{\prime }k+q}$ to states $\varepsilon_{nk}$ with emission of a phonon of energy $\hbar \omega_{\nu q}$, and mediated by the EPC matrix elements $g^{\nu }\left(nk,n^{\prime }k+q\right)$. **Figure** **4**b plots the sum of EPC strengths of valence band maximum (VBM) and conduction band maximum (CBM) locate at K point ($\lambda =\lambda_{VBMK}$ + $\lambda_{CBMK}$) for different monolayer TMDCs, which is consistent with reported theoretical values calculated by density funcitonal perturbation theory.^[^ ^32^ ^]^ Clearly, EPC strengths decrease with the atomic radii when the metal atoms are changed from Mo to W and the chalcogen atoms are changed from Te to S. This can be explained by the reduced separation between the atoms where displacements of atoms have stronger influence on electronic interactions.

To verify the relationship between $\eta_{\text{CM}}$ and EPC, the TMDCs are excited with photon energy of 2 $E_{g}$, and the carrier excess energies are distributed as $\Delta E_{\text{e/h}}∼0.7/0.3E_{g}$ (or $\Delta E_{\text{e/h}}∼0.3/0.7E_{g}$). In these cases, the effective energy for CM is $∼0.7E_{g}$. The exact values of the excess energies depend on the electronic structures of each material and is given in **Table** **1**. From Figure 4b, it is shown that there is a clear correlation between ϕ_max_ and λ for these TMDCs. In other words, the stronger EPC, the more efficient CM. As shown in Figure 4b, the strongest EPC in monolayer MoS _2_ gives rise to ∼ 7 times higher $\eta_{\text{CM}}$ compared to that in WTe _2_ with the weakest EPC. Figure 4c,d presents the change of bandgaps in each TMDCs upon excitation. Consistent with the above results, TMDC with stronger EPC results in an overall narrower average bandgap. In addition, FTs of the bandgaps reveals the characteristic oscillations come from vibrational mode A', which further confirms that the bandgap reduction and enhancement of CM are phonon‐related.

**Table 1:** Effective carrier excess energy ΔE${}_{\text{eff}}$ in six monolayer TMDCs.

	WTe_2_	MoTe_2_	WSe_2_	MoSe_2_	WS_2_	MoS_2_
ΔE${}_{\text{eff}}$ [$E_{g}$]	0.73	0.76	0.69	0.65	0.66	0.65

### 2.3 Effects of Defects on CM

**Figure 5:**
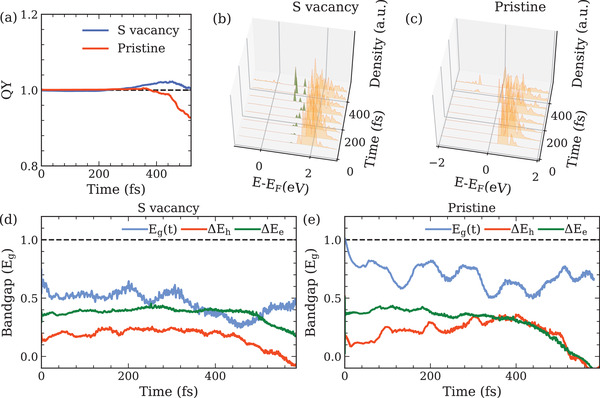
Excitation dynamics in monolayer pristine WS_2_ and WS_2_ with sulfur vacancy. Electron‐hole pairs are excited with photon energy of 1.51$E_{g}$, and carrier excess energies are distributed as $\Delta E_{\text{e/h}}=0.36/0.15E_{g}$. a) Time‐evolution of carrier generation QY of CM. Density of excited electron as a function of time and energy in b) WS_2_ with sulfur vacancy and in c) pristine WS_2_. Green fillings in (b) represent the sulfur vacancy states in the conduction bands. Time‐dependent bandgap $E_{g}\left(t\right)$, hole excess energy $\Delta E_{h}$(t) and electron excess energy $\Delta E_{e}$(t) in d) WS_2_ with sulfur vacancy and in e) pristine WS_2_.

Monolayer TMDCs, as light‐absorbing materials for solar cells, their absorption should have significant overlap with the solar spectrum and therefore be able to utilize the energy effectively for carrier generation. As Beard et al. reported, the ideal bandgap of light‐absorbing material with CM characteristics for photovoltaics should be closed to 1.0 eV.^[^ ^1^, ^4^ ^]^ Thus, it is essential to further optimize CM threshold of TMDCs to enhance their performance for photovoltaic applications. Defect engineering has been one of the key strategies of material design and defect‐assisted CM had been previously reported in amorphous silicon.^[^ ^33^ ^]^ Various defects and impurities are unavoidable during the growth processes of TMDCs. Among different defects, chalcogen vacancies are known to create sub‐gap states in TMDCs,^[^ ^34^ ^]^ which provide possibilities to manipulate the threshold energy of CM. Therefore, sulfur vacancies are introduced in our monolayer WS _2_ model, with a concentration of 3.66 $\times 10^{13}{\mathrm{cm}}^{-2}$. Due to the limitation of supercell size, the defect concentration is relatively high. Experimentally, it has been shown that the defect concentration in chemical vapor deposition synthesized MoS _2_ can be high, which is close to the concentration in our simulations.^[^ ^35^ ^]^ On the other hand, a larger supercell with a defect concentration of 9.15 × 10^12^ cm ^−2^ is constructed, in which the in‐gap defect state has essentially the same energy. Therefore, similar reduction of threshold energy is expected . As shown in Figure S13 (Supporting Information), a sulfur vacancy introduces two defect states within the bandgap, a shallow hole trap state and a deep electron trap state. The hole trap state is 0.03 eV above the VBM while the electron trap state is 0.48 eV below the CBM. As a result, the bandgap is reduced by 0.51 eV. Electron‐hole pairs are then excited with photon energy of ∼ 1.51 $E_{g}$ in both pristine WS _2_ and WS _2_ with sulfur vacancies. The carrier excess energies are distributed as $\Delta E_{\text{e/h}}$ =0.36/0.15 $E_{g}$. Here, $E_{g}$ corresponds to the bandgap of pristine WS _2_. **Figure** **5**a presents the time‐evolution of carrier generation QY after excitation. Compared with pristine WS _2_, sulfur vacancies indeed successfully lower excitation energy to only 1.51 $E_{g}$ for CM to occur. As shown in Figure 5c, for pristine WS _2_, there are two major relaxation pathways for carriers after excitation: i) electron‐hole pairs transfer the energy to higher energy states via Auger recombinationand ii) excited electrons decay to valence bands to recombine with excited holes nonradiatively. On the other hand, for WS _2_ with defects, excited electrons with sufficient energy excite another electron from valence bands to populate the defect states. This is in accordance with Figure 5d where $\Delta E_{e}\left(t\right)$ surpasses the bandgap at ∼ 250 fs and CM kicks in. In contrast, even with phonon‐induced narrowing of bandgap, the excess energies in carriers are inadequate in pristine WS _2_ for exciting additional electron‐hole pairs. The relaxation dynamics is therefore dictated by nonradiative recombination and Auger recombination only (Figure 5c,e). These results provide insights into the defect engineering for tailoring CM threshold energy to enhance performance of TMDCs for photovoltaic applications.

## 3 Conclusion

In summary, with real‐time TDDFT‐NAMD method, we investigate CM phenomenon in several monolayer TMDCs. Specifically, the role of lattice vibrations in the excited carrier dynamics is examined. It is found that upon excitation, the bandgap reduced in these monolayer TMDCs due to coupling to the out‐of‐plane A' vibrational mode. Consequently, additional electron‐hole pairs can be excited more effectively. In addition, we demonstrate chalcogen vacancies can further enhance CM by introducing defect states within the bandgap. Based on these findings, we show the possibility of triggering CM with below threshold excitation in two dimensional TMDCs. Thus, high conductivity, large absorbance and excellent CM characteristics enable monolayer TMDCs as promising materials for next‐generation photovoltaic devices.

## 4 Experimental Section

### Computational Details

First‐principles simulations of monolayer TMDCs were performed with linear combination of atomic orbital methods implemented in SIESTA.^[^ ^36^ ^]^ The core electrons were described by Troullier–Martins norm‐conserving pseudopotentials.^[^ ^37^ ^]^ The nonlocal exchange and correlation energies were treated with PBE functional.^[^ ^38^, ^39^ ^]^ Although PBE functional underestimates the bandgap of monolayer TMDCs, the phonon‐induced was considered relatively reduced bandgap. The structure was fully relaxed until the total energy variation was less than 10^−6^ eV and the residue forces were less than $5\times 10^{-3}$ eV Å^‐1^. The Brillouin Zone of unit cell was sampled by a 15 × 15 × 1 k‐mesh grid with a 250 Ry energy cutoff to obtain electronic structures. A 30 Å vacuum layer was employed to avoid the repeat image interactions. NAMD simulations were calculated based on TDDFT using the time‐dependent ab initio package (TDAP)^[^ ^40^ ^]^ with a time step of 0.02419 fs. The 3 $\sqrt{3}\times 3\sqrt{3}$ supercell was sampled at Γ point, and the initial ion velocities were obtained by the equilibrium Boltzmann–Maxwell distribution at a given temperature 300 K.

### Real‐Time TDDFT‐NAMD

The time‐dependent Schrödinger equation for coupled electron‐ion systems can be formally written as

(3) $$i\hbar \frac{\partial \Psi (r,R,t)}{\partial t}={\hat{H}}_{tot}\left(r,R,t\right)\Psi \left(r,R,t\right)$$

where $R=(R_{1},R_{2},\text{…},R_{N})$ and $r=(r_{1},r_{2},\text{…},r_{n})$ are the collective vectors for ionic and electronic positions, respectively. ${\hat{H}}_{\text{tot}}$ denotes the time‐dependent total Hamiltonian given by

(4) $$\begin{array}{ccc} {\hat{H}}_{tot}\left(r,R,t\right) & = & -\sum_{i}\frac{\hbar^{2}}{2m}\nabla_{i}-\sum_{I}\frac{\hbar^{2}}{2M_{I}}\nabla_{I}+\frac{1}{2}\sum_{i\neq j}\frac{e^{2}}{|r_{i}-r_{j}|}\\ & & +\frac{1}{2}\sum_{I\neq J}\frac{Z_{I}Z_{J}}{|R_{I}-R_{J}|}-\sum_{i,I}\frac{eZ_{I}}{|r_{i}-R_{I}|}+{\hat{V}}_{ext}\left(r,R,t\right) \end{array}$$

Here, *m* and *e* denote the electronic mass and charge, $M_{I}$ and $Z_{I}$ are the mass and charge of $I_{\text{th}}$ ion, and ${\hat{V}}_{\text{ext}}$ is the external potential. Runge‐Gross theorem indicates that the external potential for electrons ${\hat{v}}_{s}$ and ions ${\hat{V}}_{S}^{I}$ are determined by their respective densities.^[^ ^41^ ^]^

(5) $$\begin{array}{ccc} {\hat{v}}_{s}\left[\rho \right]\left(r,t\right) & = & \int \frac{\rho (r^{{}^{\prime }},t)}{|r-r^{{}^{\prime }}|}dr^{{}^{\prime }}-\sum_{I}\int \frac{Z_{I}\rho_{I}\left(r,t\right)}{|r-R_{I}|}dR_{I}+{\hat{v}}_{ext}\left(r,t\right)+{\hat{v}}_{xc}\left[\rho \right]\left(r,t\right) \end{array}$$

(6) $$\begin{array}{ccc} {\hat{V}}_{S}^{I}\left[\rho_{I}\right]\left(R_{I},t\right) & = & Z_{I}\sum_{J}\int \frac{Z_{J}\rho_{J}\left(R_{J},t\right)}{|R_{I}-R_{J}|}dR_{J}-Z_{I}\int \frac{\rho (r,t)}{|R_{I}-r|}dr\\ & & +{\hat{V}}_{ext}^{I}\left(R_{I},t\right)+{\hat{V}}_{xc}\left[\rho_{I}\right]\left(R_{I},t\right) \end{array}$$

Where $\rho \left(r,t\right)=\sum_{i}{\left|\phi_{i}\left(r,t\right)\right|}^{2}$ and $\rho_{I}\left(R_{I},t\right)={\left|\chi_{I}\left(R_{I},t\right)\right|}^{2}$.

The Ehrenfest dynamics theorem was invoked for ionic motion, in which nuclear trajectories follow Newton's second law:

(7) $$M_{I}\frac{d^{2}R_{I}\left(t\right)}{dt^{2}}=-\nabla_{I}\left[\sum_{I\neq J}\frac{Z_{I}Z_{J}}{|R_{I}-R_{J}|}-Z_{I}\int \frac{\rho (r,t)}{|R_{I}-r|}dr+{\hat{V}}_{ext}^{I}\left(R_{I},t\right)\right]$$

Note that the ion–ion exchange‐correlation functional was ignored and a sharp ionic density distribution $\rho_{I}\left(R,t\right)=\delta \left(R-R_{I}\left(t\right)\right)$ was assumed. On the other hand, the time‐dependent Kohn–Sham (TDKS) equation for the motion of electrons is as follows.

(8) $$\begin{array}{ccc} i\hbar \frac{\partial \phi_{i}\left(r,t\right)}{\partial t} & = & [-\frac{\hbar^{2}}{2m}\nabla_{i}^{2}+\int \frac{\rho (r^{\prime },t)}{|r-r^{\prime }|}dr^{\prime }-\sum_{I}\frac{Z_{I}}{|r-R_{I}|}+{\hat{v}}_{\mathrm{ext}}\left(r,t\right)\\ & & +\;{\hat{v}}_{\mathrm{xc}}\left[\rho \right]\left(r,t\right)]\phi_{i}\left(r,t\right) \end{array}$$

The electronic subsystem describes quantum mechanically within TDDFT, while the nuclear subsystem evolves along with classical Newton's trajectories with the forces obtained from Ehrenfest theorem.^[^ ^42^, ^43^ ^]^ Ehrenfest dynamics is known for violating the principle of detailed balance overestimate vibronic coherence. Consequently, the heat dissipation from excited carriers to nuclei was suppressed and for a long simulation time, the mean vibrational energies exceeded the thermodynamical limit. In contrast, surface hopping dynamics takes care of detailed balance by carefully assigning hops between states. To tackle this problem, correction for decoherence and detailed balance in Ehrenfest dynamics was proposed.^[^ ^44^ ^]^ It was expected that with these corrections, CM process would occur earlier in the simulations since this was triggered by nuclear vibrations, as shown in Figure 3. Meanwhile, lowering of threshold energy for carrier multiplication should still hold since similar bandgap reduction during the time evolution should be observed. In addition, to avoid the incorrect long time limit, the simulations were limited to early stage of carrier dynamics within several hundred femtoseconds after photo excitation.

### EPC Parameters

The calculations of EPC parameters λ was based on Kohn–Sham DFT implemented in SIESTA^[^ ^36^ ^]^ together with INELEASTICA.^[^ ^45^ ^]^ In the limitation of low temperature, phonon absorption is suppressed and Eliashberg function α^2^F is written as

(9) $$\begin{array}{ccc} \alpha^{2}F_{nk}^{E}\left(\omega \right) & = & \sum_{\nu q}\sum_{n^{\prime }}{\left|g^{\nu }\left(nk,n^{\prime }k+q\right)\right|}^{2}\\ & & \times \delta \left(\varepsilon_{n^{\prime }k+q}-\varepsilon_{nk}-\hbar \omega_{\nu q}\right)\delta \left(h\omega -h\omega_{\nu q}\right) \end{array}$$

where the summations include all electron scattering from $\varepsilon_{n^{\prime }k+q}$ states to $\varepsilon_{nk}$ states with emission of a phonon with energy of $\hbar \omega_{\nu q}$. This transition process was mediated by EPC matrix elements $g^{\nu }\left(nk,n^{\prime }k+q\right)$. The EPC parameter $\lambda_{nk}$ is defined as

(10) $$\lambda_{nk}=2\int d\omega \frac{\alpha^{2}F_{nk}^{E}\left(\omega \right)}{\omega }$$

For monolayer TMDCs, VBM and CBM of monolayer TMDCs located at **K** point, thus **q** was considered that was around **K** point with a distance of 0.1 Å^−1^ in reciprocal space to satisfy energy conservation.

## Conflict of Interest

The authors declare no conflict of interest.

## Supporting information

Supporting Information

## Data Availability

The data that support the findings of this study are available from the corresponding author upon reasonable request.
