# Supplementary material for: Carrier Multiplication in Transition Metal Dichalcogenides Beyond Threshold Limit
Source: Adv Sci (Weinh). 2022 Sep 7;9(31):2203400. doi: 10.1002/advs.202203400 (PMC9631089; doi:10.1002/advs.202203400)
Supplement: Supplementary file 1 — Supporting Information [file ADVS-9-2203400-s001.pdf]

ADVANCED SCIENCE
Open Access

Supporting Information

for *Adv. Sci.*, DOI 10.1002/advs.202203400

Carrier Multiplication in Transition Metal Dichalcogenides Beyond Threshold Limit

Yuxiang Liu, Thomas Frauenheim and ChiYung Yam**

Carrier Multiplication in Transition Metal Dichalcogenides Beyond Threshold Limit

Yuxiang Liu,[†] Thomas Frauenheim,[*,†,‡,¶] and ChiYung Yam[*,§,‖]

†*Bremen Center for Computational Materials Science, University of Bremen, Am Fallturm 1, 28359 Bremen, Germany*

‡*Beijing Computational Science Research Center, Haidian District, Beijing, 100193, China*

¶*Shenzhen JL Computational Science and Applied Research Institute, Shenzhen, 518109, China*

§*Shenzhen Institute for Advanced Study, University of Electronic Science and Technology of China, Shenzhen, 518000, China*

‖*Hong Kong Quantum AI Lab Limited, Hong Kong, China*

E-mail: thomas.frauenheim@bccms.uni-bremen.de; yamcy@uestc.edu.cn

Supporting Information

Electronic Structures of monolayer TMDCs

To investigate carrier multiplication (CM) phenomena in monolayer transition metal dichalcogenides (TMDCs), we build a $3\sqrt{3} \times 3\sqrt{3}$ supercell with 81 atoms shown in Figure S1(a). Van der Waals (vdW) layered TMDCs, represented by MX_2 (M = Mo, W; X = Te, Se, S), exhibit indirect-to-direct bandgap transition when exfoliated from bulk to monolayer. As shown in Figure S2, the six different monolayer TMDCs are all direct semiconductors, and the bandgap increases as the anions change from sulfur to tellurium and cations change from

molybdenum to tungsten owing to the decrease in electronegativity and increase of ionic radii.

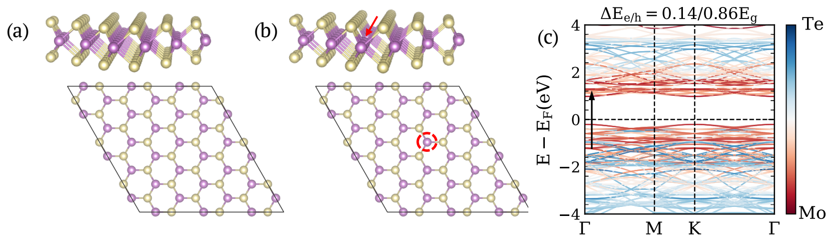

Figure S 1: Side view and top view of the $3\sqrt{3} \times 3\sqrt{3}$ supercell of (a) pristine monolayer MX_2, (b) monolayer MX_2 with chalcogen vacancy defect. The yellow and purple spheres represent chalcogen and transition metal atoms, respectively. Chalcogen vacancy defect is highlighted with red arrow and red cycles at side and top views. (c) Electron-hole pairs excited with photon energy of $2E_g$. The hole and the electron excess energy are $0.86E_g$ and $0.14E_g$, respectively.

Excitation Dynamics in monolayer $MoTe_2$

To investigate CM in monolayer $MoTe_2$, electron-hole pairs with energy of $2E_g$ are created, in which hole excess energy ΔE_h is $0.86E_g$ and electron excess energy ΔE_e is $0.14E_g$ (Figure S1(c)). As discussed in the main text, the phonon-induced reduction of bandgap drives the onset of CM, in which excited holes have sufficient energy to scatter additional electron-hole pairs in the system. Compared with experimental observation, where the CM conversion efficiency η_{CM} can be as high as 94∼99%,[1,2] the carrier generation quantum yield (QY) in our simulations is inefficient. The origin of the relatively low CM conversion efficiency in our calculations is as follows. Firstly, twelve electron-hole pairs are excited in monolayer $MoTe_2$, with a concentration of $3.58\times10^{-14}cm^{-2}$ which is larger than that in experiment work. Thus, an efficient Auger recombination is resulted. As shown in Figure S3(a)-(b), partial excited holes and electrons populate higher energy states, which is the fingerprint of Auger process. This decay dynamics can be fitted by exponential decay, with a lifetime of ∼1.98 ps. This can be attributed to Auger trapping free carriers which is detected on 1-2 ps time scale in TMDCs.[3–6] Furthermore, excitation dynamics of monolayer $MoTe_2$ with one electron-hole

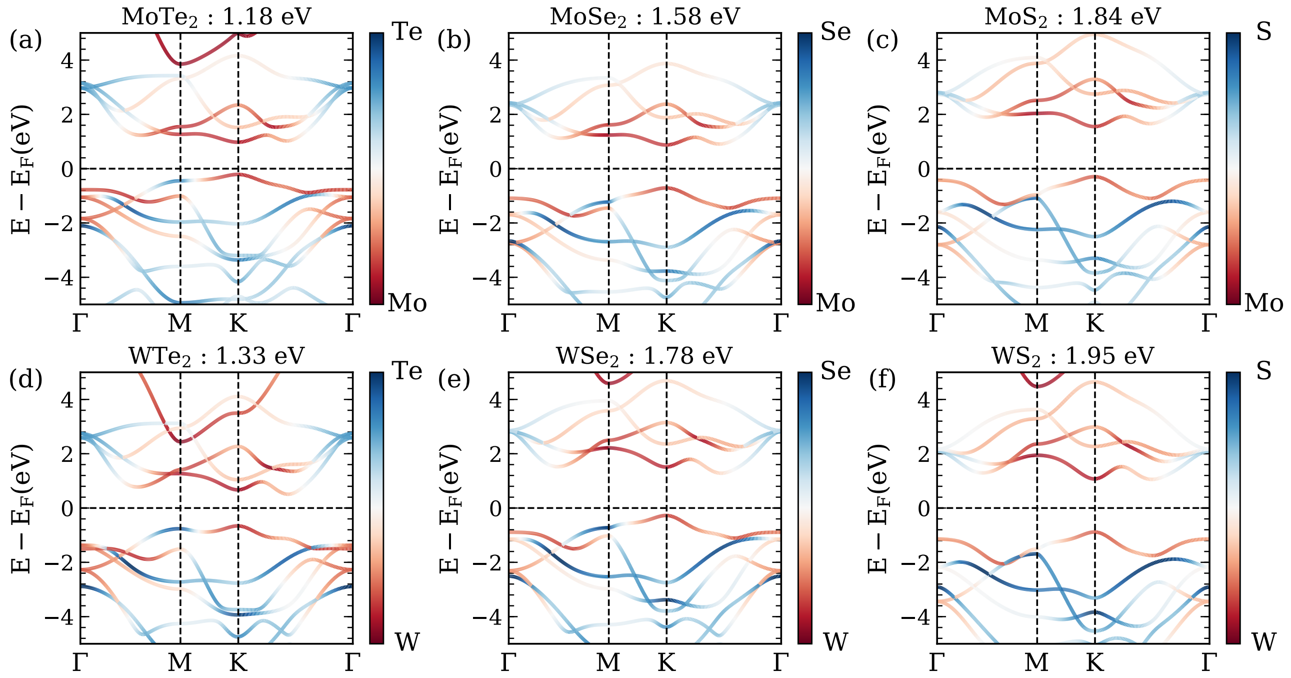

Figure S 2: Band structure of monolayer (a) $MoTe_2$, (b) $MoSe_2$, (c) MoS_2, (d) WTe_2, (e) WSe_2 and (f) WS_2. The dashed lines show the Fermi level E_F.

pair is simulated, as shown in Figure S4. The maximum carrier generation QY equals to 2, and conversion efficiency η_{CM} reaches up to 100% which is consistent with experimental observations.[1,2] In addition, surface/defect trapping which effectively competes with Auger process also make contributions to the higher conversion efficiency in experiments. Apart from Auger recombination, monolayer crystal structure accelerates charge carrier nonradiative recombination. Figure S3(a)-(b) present that excited hole orbitals partially occupy conduction band states while excited electrons populate valence band states, corresponding to the nonradiative recombination. Generally, the charge carrier nonradiative recombination rate in monolayer TMDCs is on the order of several hundreds of picoseconds.[7,8]

For further analysis the role of EPC in CM process, carriers are excited in monolayer $MoTe_2$ with energy of $2E_g$ at 77 K, 300 K and 500 K, respectively. Excitations with two different excess energy distribution are simulated ($\Delta E_{e/h}$=0.14/0.86E_g and $\Delta E_{e/h}$=0.50/0.50E_g). As discussed in the main text, for asymmetric electron-hole pair, CM phenomenon is observed at all three temperatures. It is found that increasing temperature has a positive effect

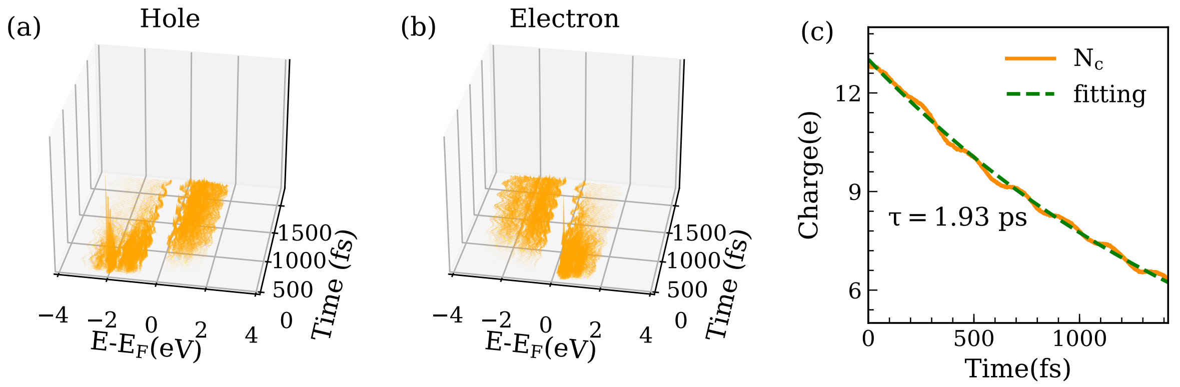

Figure S 3: Excitation dynamics of monolayer $MoTe_2$. Twelve electron-hole pairs are excited with carrier excess energy of $\Delta E_{e/h}$=0.14/0.86E_g Density of excited (a) hole and (b) electron as a function of time and energy. (c) Decay dynamics of carrier after reaching maximum carrier generation. The carrier lifetime is fitted with an exponential function and lifetime is found to be 1.93 ps.

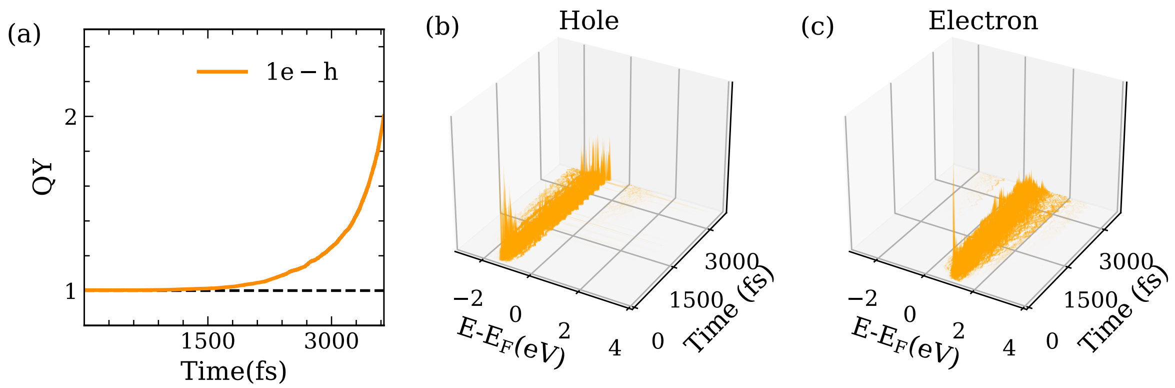

Figure S 4: Excitation dynamics in monolayer $MoTe_2$. One electron-hole pair are generated, and carrier excess energy are distributed as $\Delta E_{e/h}$=0.14/0.86E_g. (a) Time-evolution of carrier generation QY. Density of excited (a) hole and (b) electron as a function of time and energy.

on improving carrier generation QY. Compared with 77 K, the CM conversion efficiency η_{CM} is about doubled at 500 K. With a higher temperature, ionic vibrations are more intense. This results in a narrower bandgap and consequently more efficient carrier generation QY. As shown in Figure S5 and Figure 1, from 77 K to 500 K, the reduction of bandgap increasing from 28% to 64%. If keep increasing temperature, semicondutor-to-metal transitions may eventually kick in. In the case of carriers with excess energies of $\Delta E_{e/h}$=0.5/0.5E$_g$, CM phenomenon is only observed in 500 K among the three cases. Consistently, Figure S6(a) shows that the electron excess energy ΔE_e(t) is beyond E$_g$(t) at about 100 fs. In contrast, E$_g$(t) is always larger than $\Delta E_{e/h}$(t) after excitation in the case of 77 K and 300 K. Therefore, it is possible to break the threshold limit for CM via coupling to phonons.

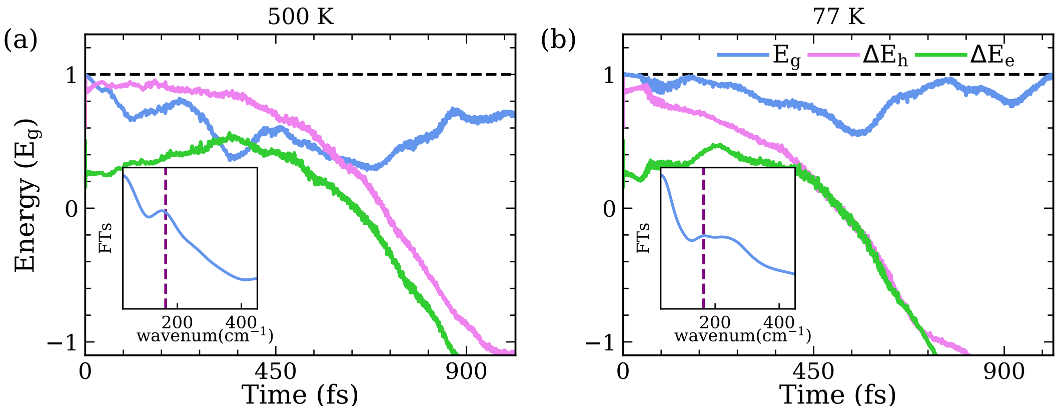

Figure S 5: Excitation dynamics in monolayer $MoTe_2$ with carrier excess energy of $\Delta E_{e/h} = 0.14/0.86 Eg$. Electron-hole pairs are excited at different temperatures. Comparison of E$_g$(t), ΔE_e(t) and ΔE_h(t) at excitation temperatuer of (a) 500K and (b) 77 K. Inset: FTs of time-dependent bandgap $E_g(t)$. Vertical dashed lines represent phonon mode A' with vibrational frequency of 163.7 cm^{-1}.

Lattice vibrational modes in monolayer TMDCs

To verify the relationship between nuclear motions and electronic structures, we simulate lattice vibrational modes in monolayer TMDCs. The phonon disperion and ionic vibrations of four optical phonon modes are shown in Figure2 and Figure S7-Figure S11. These calculated vibration frequencies coincide with experimental measurements.[9–13] Similar to monolayer

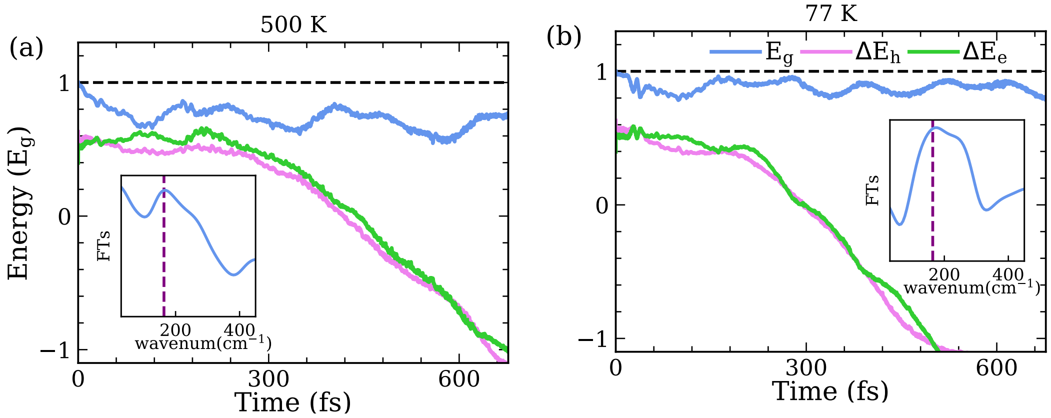

Figure S 6: Excitation dynamics in monolayer $MoTe_2$ with carrier excess energy of $\Delta E_{e/h} = 0.50/0.50Eg$. Electron-hole pairs are excited at different temperatures. Comparison of E_g(t), ΔE_e(t) and ΔE_h(t) at excitation temperatuer of (a) 500K and (b) 77 K. Inset: FTs of time-dependent bandgap $E_g(t)$. Vertical dashed lines represent phonon mode A' with vibrational frequency of 163.7 cm^{-1}.

$MoTe_2$, all four optical phonons, including A", E', A' and E" modes, significantly change the electronic structures in the other five monolayer TMDCs, leading to a narrowing of bandgap.

To further demonstrate the role of bandgap in CM process, we apply strain along the lattice parameter directions in monolayer $MoTe_2$, with a range of -3% $\sim$ +3%. The inset of Figure S12 shows that tensile and compressive strains both narrow the bandgap of $MoTe_2$ which is expected to enhance QY of CM. As shown in Figure S12, +3% strain improve QY of CM slightly, with a value of $\sim$1.08, while QY in the case with -3% strain gives rise up to $\sim$1.12, increasing around 30%. This results indicate that the narrower bandgap of monolayer TMDC indeed have positive effect in improving CM performance, which can be manipulated by nuclear vibrations.

Chalcogen vacancy defect

To optimize the electronic structures of TMDCs to enhance their performance for photovoltaic applications, a sulfur vacancy is introduced to the $3\sqrt{3} \times 3\sqrt{3}$ supercell of monolayer WS_2 (Figure S1(b)), corresponding to a defect concentration of 3.66$\times$ 10^{13}cm^{-2}. As shown in Figure S13, the calculated bandgap of pristine monolayer WS_2 is 1.95 eV, which is con-

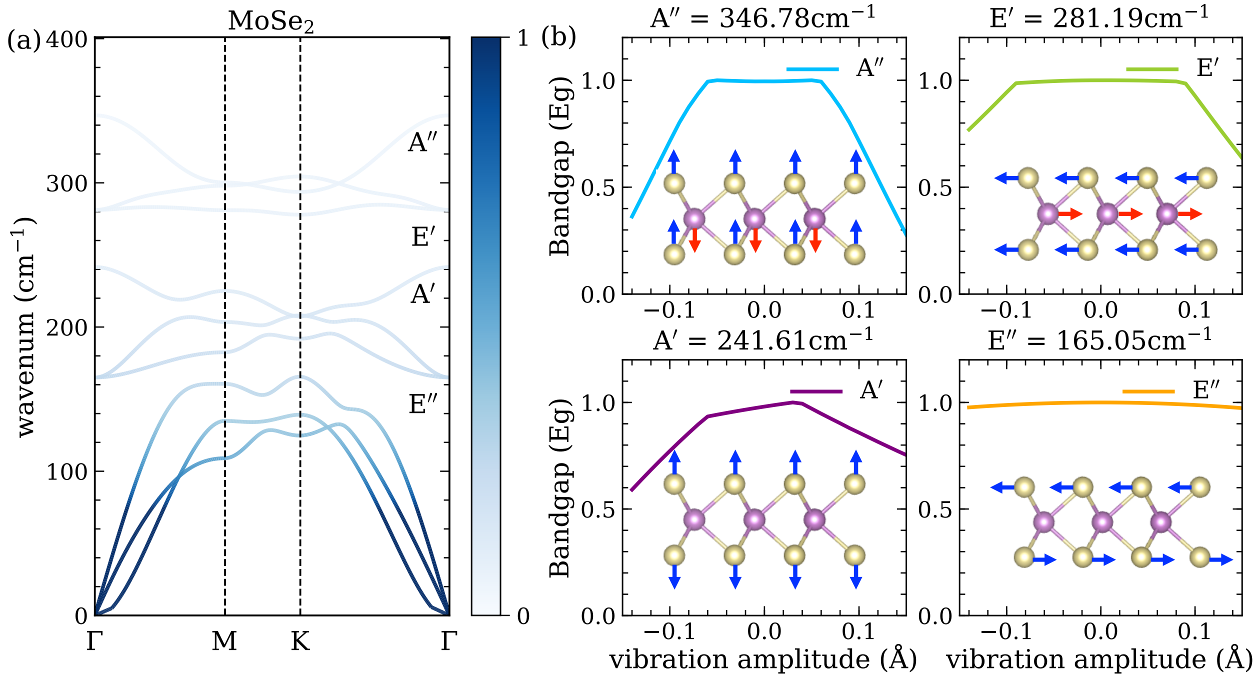

Figure S 7: Lattice vibrational modes in monolayer $MoSe_2$. (a) Phonon dispersion of monolayer $MoSe_2$. The colormap indicates the phonon occupation number which is determined by the Bose-Einstein distribution at 300K. (b) The bandgap as a function of vibration amplitude of four optical phonon in $MoSe_2$. The insets show the corresponding lattice vibrations for optical phonons at Γ point.

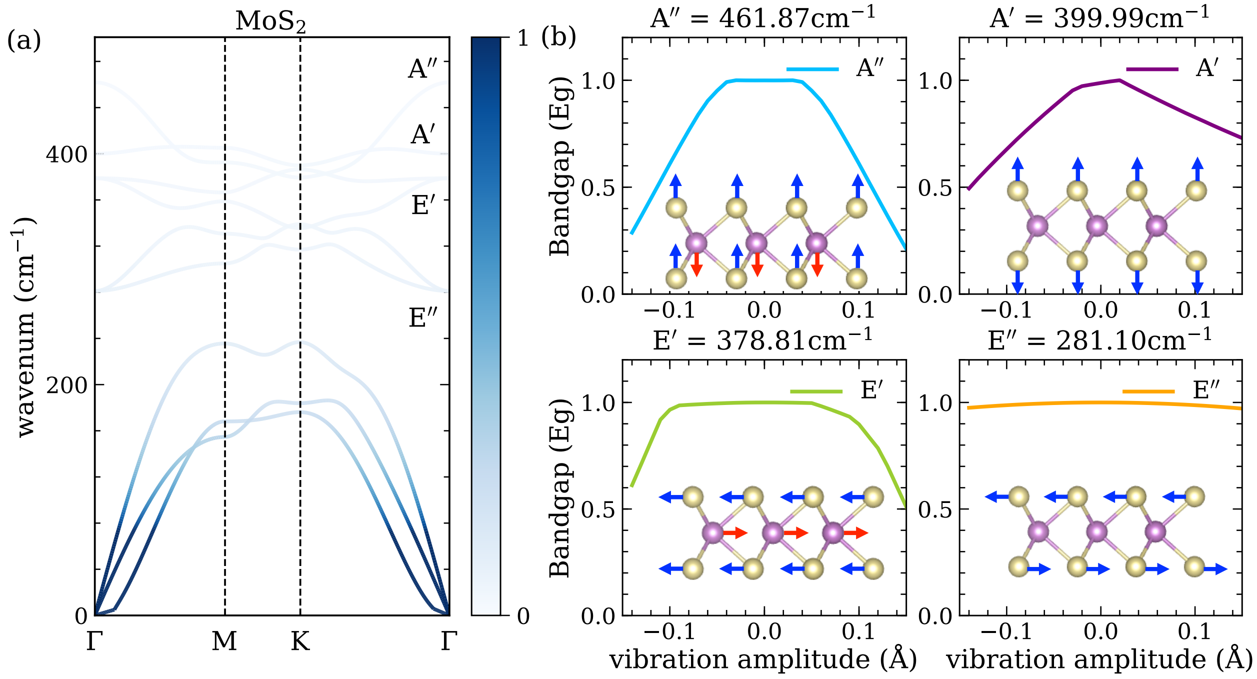

Figure S 8: Lattice vibrational modes in monolayer MoS_2. (a) Phonon dispersion of monolayer MoS_2. The colormap indicates the phonon occupation number which is determined by the Bose-Einstein distribution at 300K. (b) The bandgap as a function of vibration amplitude of four optical phonon in MoS_2. The insets show the corresponding lattice vibrations for optical phonons at Γ point.

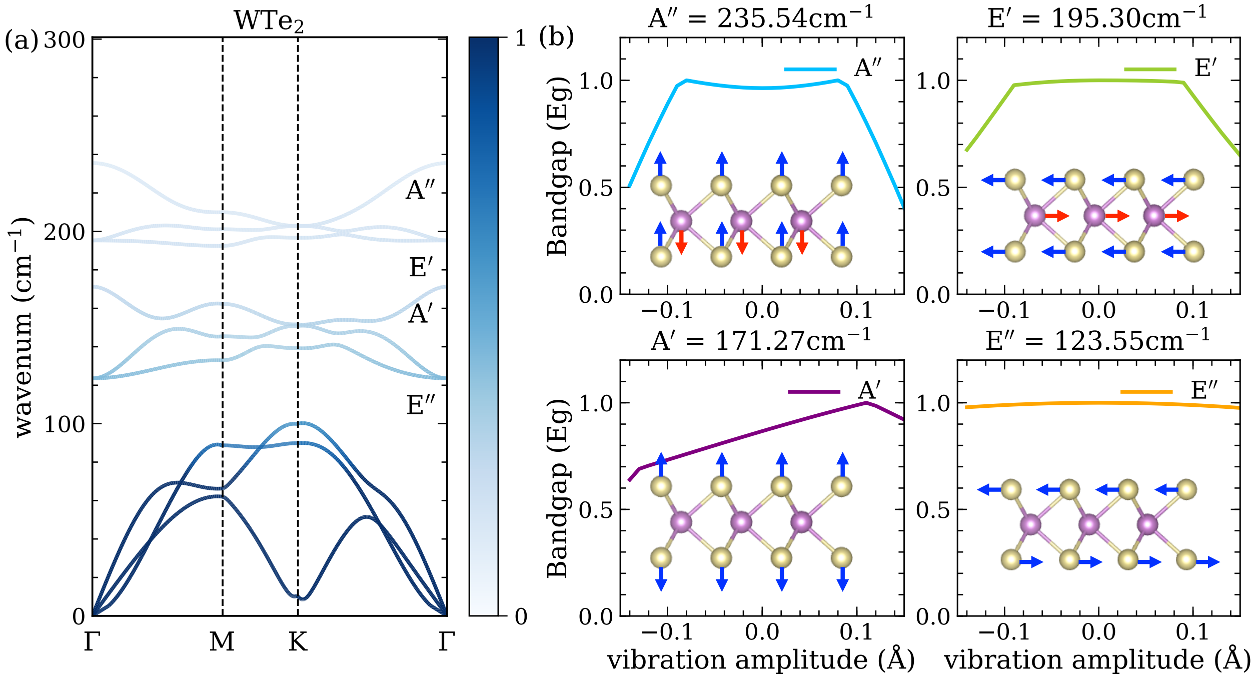

Figure S 9: Lattice vibrational modes in monolayer WTe_2. (a) Phonon dispersion of monolayer WTe_2. The colormap indicates the phonon occupation number which is determined by the Bose-Einstein distribution at 300K. (b) The bandgap as a function of vibration amplitude of four optical phonon in WTe_2. The insets show the corresponding lattice vibrations for optical phonons at Γ point.

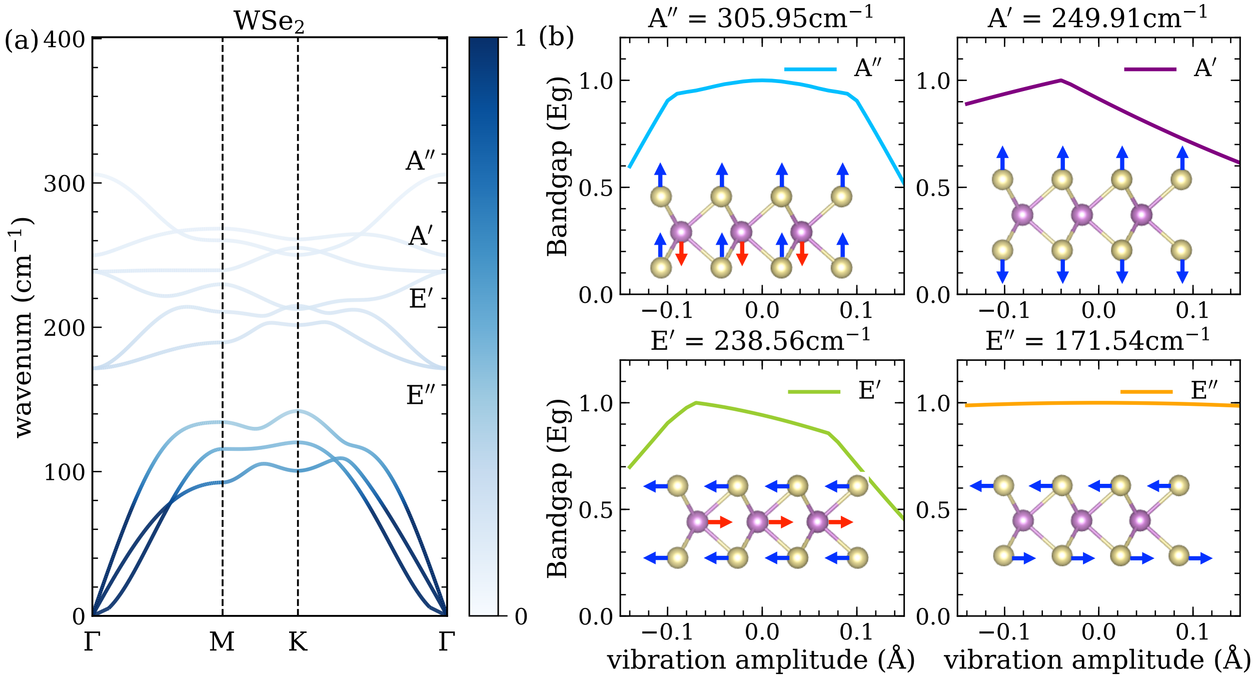

Figure S 10: Lattice vibrational modes in monolayer WSe_2. (a) Phonon dispersion of monolayer WSe_2. The colormap indicates the phonon occupation number which is determined by the Bose-Einstein distribution at 300K. (b) The bandgap as a function of vibration amplitude of four optical phonon in WSe_2. The insets show the corresponding lattice vibrations for optical phonons at Γ point.

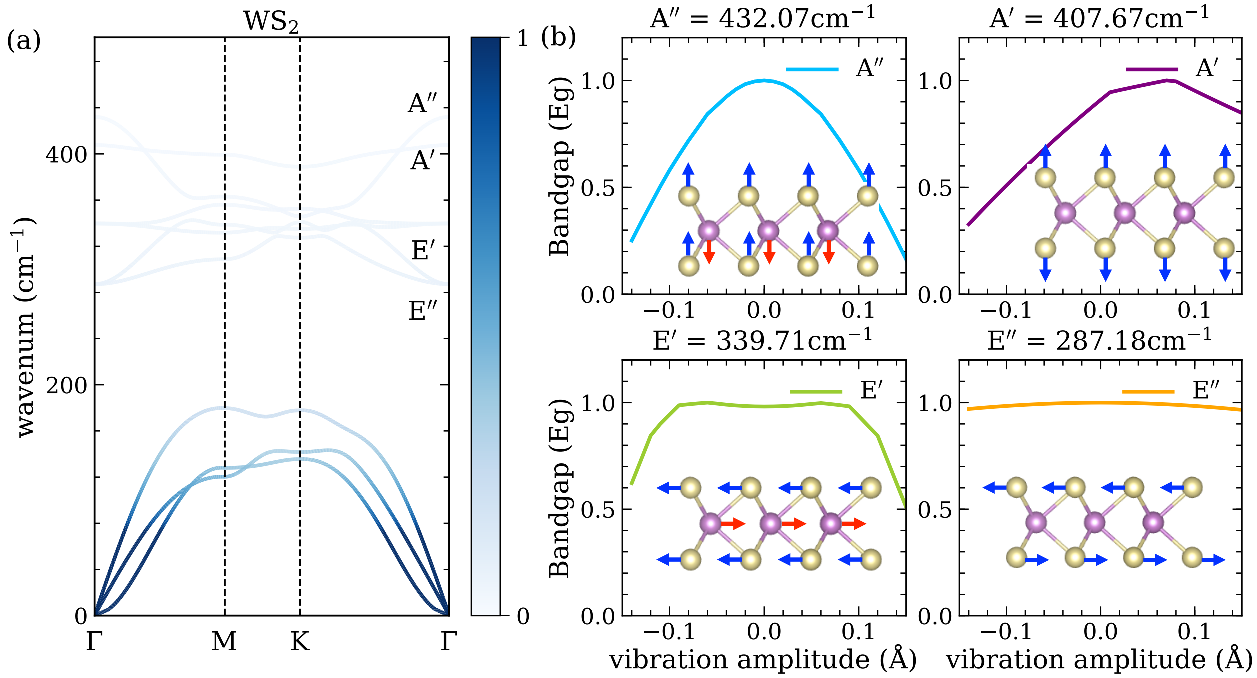

Figure S 11: Lattice vibrational modes in monolayer WS_2. (a) Phonon dispersion of monolayer WS_2. The colormap indicates the phonon occupation number which is determined by the Bose-Einstein distribution at 300K. (b) The bandgap as a function of vibration amplitude of four optical phonon in WS_2. The insets show the corresponding lattice vibrations for optical phonons at Γ point.

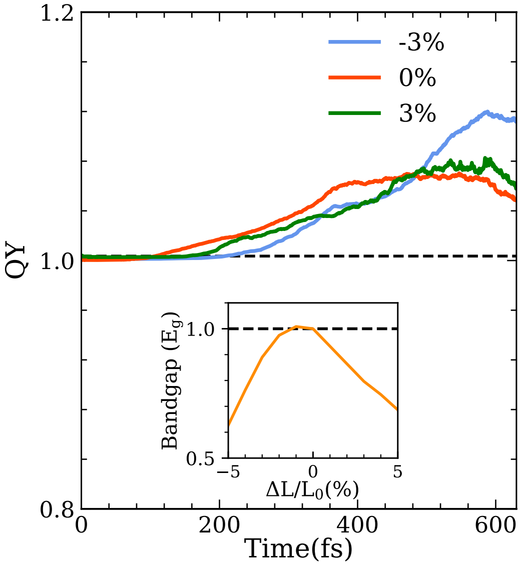

Figure S 12: Excitation dynamics in monolayer $MoTe_2$ with applied strain. Electron-hole pairs are excited with carrier excess energy of $\Delta E_{e/h} = 0.14/0.86E_g$. Inset: The bandgap of monolayer $MoTe_2$ under tensile strain.

sistent with previously reported values.[14] The sulfur vacancies create two defect states, one is shallow hole trap states which reduces bandgap by a value of 0.03 eV, and the other is deep electron trap states that is 0.48 eV below the CBM. Thus, sulfur vacancies lower the bandgap of monolayer WS_2 by a vaule of ~0.51 eV.

Carriers are excited in pristine monolayer WS_2 and monolayer WS_2 with sulfur vacancy by photons with energy of 1.51 E_g. The carrier excess energy are the same in two cases, with a value of $\Delta E_{e/h}$=0.36/0.15E_g. Compared with pristine monolayer WS_2, with phonon-assisted modulation and defect states, CM phenomenon is observed in monolayer WS_2 with sulfur vacancy, and CM conversion efficiency is ~2.37%. As shown in Figure5(b)-(c) and Figure S14, the deep electron defect states play an important role in the relaxation dynamics, in which excited holes with sufficient energy scatter additional electrons from occupied valence bands to these deep trap states. Eventually, CM process prevails over Auger decay and nonradiative recombination.

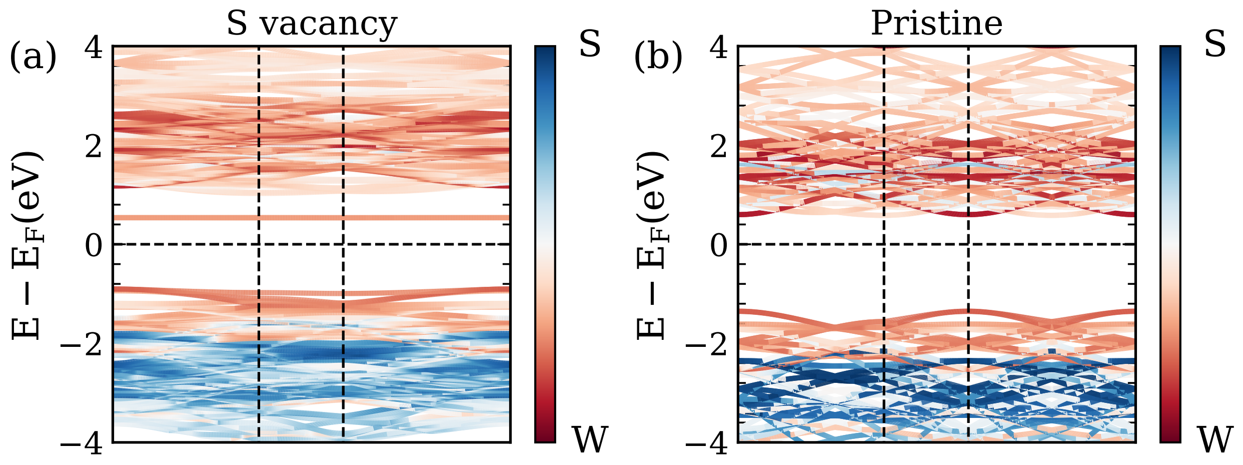

Figure S 13: Bandstructures of $3\sqrt{3} \times 3\sqrt{3}$ supercell of (a) monolayer WS_2 with S vacancy, and (b) pristine monolayer WS_2. The dashed lines indicate the Fermi levels.

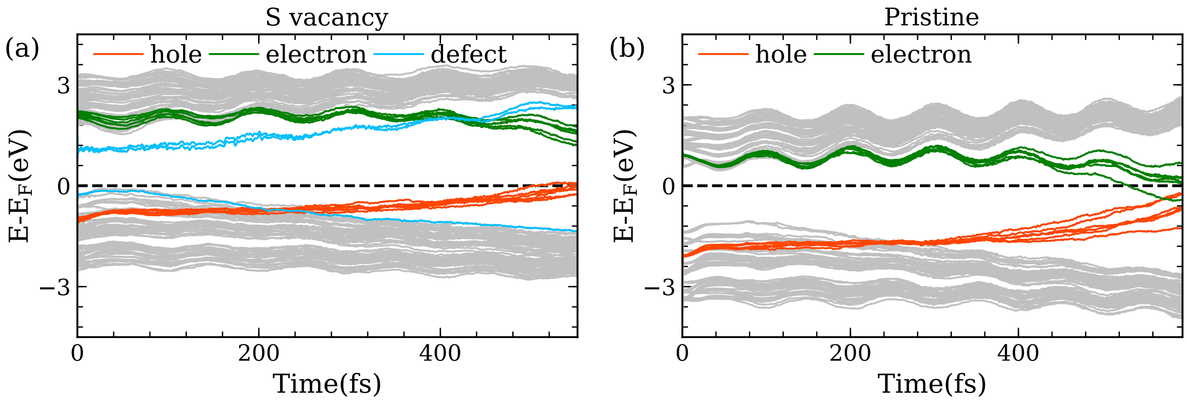

Figure S 14: Time-evolution of energy states after excitation in (a) monolayer WS_2 with sulfur vacancy, and (b) pristine mononlayer WS_2. The blue, red and green lines aredefect states, excited hole and electron states, respectively. The dashed lines indicate the Fermi levels.

References

(1) Kim, J.-H.; Bergren, M. R.; Park, J. C.; Adhikari, S.; Lorke, M.; Frauenheim, T.; Choe, D.-H.; Kim, B.; Choi, H.; Gregorkiewicz, T., et al. Carrier multiplication in van der Waals layered transition metal dichalcogenides. *Nature Communications* **2019**, *10*, 1–9.

(2) Zheng, W.; Bonn, M.; Wang, H. I. Photoconductivity Multiplication in Semiconducting Few-Layer $MoTe_2$. *Nano Letters* **2020**, *20*, 5807–5813.

(3) Wang, H.; Zhang, C.; Rana, F. Ultrafast dynamics of defect-assisted electron–hole recombination in monolayer MoS_2. *Nano Letters* **2015**, *15*, 339–345.

(4) Wang, H.; Strait, J. H.; Zhang, C.; Chan, W.; Manolatou, C.; Tiwari, S.; Rana, F. Fast exciton annihilation by capture of electrons or holes by defects via Auger scattering in monolayer metal dichalcogenides. *Physical Review B* **2015**, *91*, 165411.

(5) Kar, S.; Su, Y.; Nair, R. R.; Sood, A. Probing photoexcited carriers in a few-layer MoS_2 laminate by time-resolved optical pump–terahertz probe spectroscopy. *ACS Nano* **2015**, *9*, 12004–12010.

(6) Li, L.; Lin, M.-F.; Zhang, X.; Britz, A.; Krishnamoorthy, A.; Ma, R.; Kalia, R. K.; Nakano, A.; Vashishta, P.; Ajayan, P., et al. Phonon-suppressed auger scattering of charge carriers in defective two-dimensional transition metal dichalcogenides. *Nano Letters* **2019**, *19*, 6078–6086.

(7) Korn, T.; Heydrich, S.; Hirmer, M.; Schmutzler, J.; Schüller, C. Low-temperature photocarrier dynamics in monolayer MoS_2. *Applied Physics Letters* **2011**, *99*, 102109.

(8) Shi, H.; Yan, R.; Bertolazzi, S.; Brivio, J.; Gao, B.; Kis, A.; Jena, D.; Xing, H. G.; Huang, L. Exciton dynamics in suspended monolayer and few-layer MoS_2 2D crystals. *ACS Nano* **2013**, *7*, 1072–1080.

(9) Han, H.-V.; Lu, A.-Y.; Lu, L.-S.; Huang, J.-K.; Li, H.; Hsu, C.-L.; Lin, Y.-C.; Chiu, M.-H.; Suenaga, K.; Chu, C.-W., et al. Photoluminescence enhancement and structure repairing of monolayer $MoSe_2$ by hydrohalic acid treatment. *Acs Nano* **2016**, *10*, 1454–1461.

(10) Wang, Y.; Cong, C.; Qiu, C.; Yu, T. Raman spectroscopy study of lattice vibration and crystallographic orientation of monolayer MoS_2 under uniaxial strain. *Small* **2013**, *9*, 2857–2861.

(11) Oliver, S. M.; Young, J.; Krylyuk, S.; Reinecke, T. L.; Davydov, A. V.; Vora, P. M. Valley phenomena in the candidate phase change material $WSe_{2(1-x)}Te_{2x}$. *Communications Physics* **2020**, *3*, 1–10.

(12) Terrones, H.; Corro, E. D.; Feng, S.; Poumirol, J.; Rhodes, D.; Smirnov, D.; Pradhan, N.; Lin, Z.; Nguyen, M.; Elías, A., et al. New first order Raman-active modes in few layered transition metal dichalcogenides. *Scientific Reports* **2014**, *4*, 1–9.

(13) Wang, F.; Kinloch, I. A.; Wolverson, D.; Tenne, R.; Zak, A.; O'Connell, E.; Bangert, U.; Young, R. J. Strain-induced phonon shifts in tungsten disulfide nanoplatelets and nanotubes. *2D Materials* **2016**, *4*, 015007.

(14) Gusakova, J.; Wang, X.; Shiau, L. L.; Krivosheeva, A.; Shaposhnikov, V.; Borisenko, V.; Gusakov, V.; Tay, B. K. Electronic properties of bulk and monolayer TMDs: theoretical study within DFT framework (GVJ-2e method). *Physica Status Solidi (a)* **2017**, *214*, 1700218.
